# Supplementary material for: A Critical View of the Application of the APEX Software (Aqueous Photochemistry of Environmentally-Occurring Xenobiotics) to Predict Photoreaction Kinetics in Surface Freshwaters
Source: Molecules. 2019 Dec 18;25(1):9. doi: 10.3390/molecules25010009 (PMC7017383; doi:10.3390/molecules25010009)
Supplement: Supplementary file 1 [file molecules-25-00009-s001.zip › 3.2.4_gcc-4.4.0.zip!/3.2.4_gcc-4.4.0/3.2.4_gcc-4.4.0/doc/octave/html/interpreter/Process-ID-Information.html]

Process ID Information - Untitled


Next: Environment Variables,
Previous: Controlling Subprocesses,
Up: System Utilities


---

### 34.6 Process, Group, and User IDs

— Built-in Function: pgid = **getpgrp** ()  
> Return the process group id of the current process.

— Built-in Function: pid = **getpid** ()  
> Return the process id of the current process.

— Built-in Function: pid = **getppid** ()  
> Return the process id of the parent process.

— Built-in Function: euid = **geteuid** ()  
> Return the effective user id of the current process.

— Built-in Function: uid = **getuid** ()  
> Return the real user id of the current process.

— Built-in Function: egid = **getegid** ()  
> Return the effective group id of the current process.

— Built-in Function: gid = **getgid** ()  
> Return the real group id of the current process.
